# Comparative RNA-Seq analysis reveals genes associated with masculinization in female *Cannabis sativa*

**DOI:** 10.1007/s00425-020-03522-y

**Published:** 2021-01-04

**Authors:** Ayelign M. Adal, Ketan Doshi, Larry Holbrook, Soheil S. Mahmoud

**Affiliations:** 1 Department of Biology, The University of British Columbia, Kelowna, BC Canada; 2 Zyus Life Sciences Inc., 204-407 Downey Rd, Saskatoon, SK Canada

**Keywords:** Cannabis, Flower sex, Differential expression, Male biased-genes, RNA-seq, Silver thiosulfate

## Abstract

**Main conclusion:**

Using RNA profiling, we identified several silver thiosulfate-induced genes that potentially control the masculinization of female *Cannabis sativa* plants.

**Abstract:**

Genetically female *Cannabis sativa* plants normally bear female flowers, but can develop male flowers in response to environmental and developmental cues. In an attempt to elucidate the molecular elements responsible for sex expression in *C. sativa* plants, we developed genetically female lines producing both female and chemically-induced male flowers. Furthermore, we carried out RNA-Seq assays aimed at identifying differentially expressed genes responsible for male flower development in female plants. The results revealed over 10,500 differentially expressed genes, of which around 200 potentially control masculinization of female cannabis plants. These genes include transcription factors and other genes involved in male organ (i.e., anther and pollen) development, as well as genes involved in phytohormone signalling and male-biased phenotypes. The expressions of 15 of these genes were further validated by qPCR assay confirming similar expression patterns to that of RNA-Seq data. These genes would be useful for understanding predisposed plants producing flowers of both sex types in the same plant, and help breeders to regulate the masculinization of female plants through targeted breeding and plant biotechnology.

**Supplementary Information:**

The online version contains supplementary material available at 10.1007/s00425-020-03522-y.

## Introduction

*Cannabis sativa* (Cannabaceae) is a dioecious species with male and female individuals that produce unisexual flowers (Rana and Choudhary 2010). Male flowers develop in hanging panicles within the male inflorescence, in which each flower includes an androecium of five short-stalked stamens that are enclosed by a perianth of five sepals. Female flowers form a raceme within the female inflorescence that develops at the apex, as well as the axils of leaves and lateral branches. The female flower is composed of a green bract of modified leaves that completely encloses the perianth and the uniloculate ovary. The ovary has a short style and produces a bifid stigma at maturity (Mohan Ram and Nath 1964).

The therapeutic properties of cannabis plants are mainly associated with female flowers (buds), which accumulate large amounts of several cannabinoids in stalked capitate and sessile trichomes, including Δ9-tetrahydrocannabinolic acid (THCA) and cannabidiolic acid (CBDA) (van Bakel et al. 2011; Thomas and ElSohly 2016; Bernstein et al. 2019). The floral tissues also produce a suite of over 50 mono- and sesquiterpenes, which impart the characteristic aroma and scent to cannabis plants, and account for at least some of the medicinal properties of cannabis extracts. Given their importance as the main sites for the production of cannabinoids and terpenes, there is substantial interest in understanding the molecular mechanisms that control flower development and sex differentiation in cannabis plants.

The molecular mechanisms of flower formation and floral pattern development have been extensively studied in the bisexual model plant species *Arabidopsis thaliana*, as well as some other model plants, e.g., petunia (Kater et al. 1998; Causier et al. 2010; Litt and Kramer 2010). Based on these studies, flower development in angiosperms follows the ABC(DE) model (Searle and Coupland 2004; Causier et al. 2010; Bouché et al. 2016; Irish 2017), which explains the roles of different classes of genes (A, B, C, D, and E genes) involved in the development of various floral parts in four concentric whorls of sepals, petals, stamens and carpels (Causier et al. 2010; Bouché et al. 2016). For instance, the development of sepals is controlled by A + E genes, petals by A + B + E, stamens by B + C + E, carpels by C + E, and ovules by C + D + E (Bouché et al. 2016). The key representative genes include *APETALA1 (AP1)* and *APETALA2 (AP2)* under A-class, *APETALA3 (AP3)* and *PISTILLATA (PI)* under B-class, *AGAMOUS (AG)* under C-class, *SHATTERPROOF (SHP)* and *SEEDSTICK (STK)* under D-class, and *SEPALLATA (SEP)* under E-class. Silencing and/or overexpression of these genes can have deleterious homeotic effects, including elimination or conversion of floral parts in *Arabidopsis* (Kram et al. 2009; Bouché et al. 2016; Wils and Kaufmann 2017). Homologs of different A-, B-, C-, D- and E-class genes with similar or identical functions have also been identified in non-model plants, including lavender (Litt and Kramer 2010; Gao et al. 2017; Wells et al. 2020).

Sex determination in the cannabis flower is controlled primarily at the genetic level. In this diploid species (2*n* = 20), the chromosome set is composed of nine pairs of autosomes and one pair of sex chromosomes, XX for female and XY for male plants (Moliterni et al. 2004; van Bakel et al. 2011; Divashuk et al. 2014). Factors other than genetic makeup (e.g., growth conditions, photoperiod, plant hormones, and certain chemicals) can also influence the sex of the cannabis flower, and induce the development of male flowers in female plants (Grant et al. 1994). This poses a problem for farmers growing drug-type female plants for cannabinoids, since male flowers with fewer trichomes accumulate limited amounts of cannabinoids. As well, the emergence of male flowers leads to the fertilization of female flowers and seed production, lowering cannabinoid yield for medical marijuana, while the rapidly growing hemp farming industry requires sex determination genetics for seed and fiber quality. There is, therefore, an interest in understanding the molecular basis of male flower development in cannabis. In this context, the application of silver thiosulfate or silver nitrate has a masculinizing effect, and can induce the development of male flowers in female plants (Mohan Ram and Sett 1980; Ram and Sett 1982; Devani et al. 2017; Li et al. 2017). In this study, we treated genetically female plants with silver thiosulfate (Ag_2_S_2_O_3_) complex to induce male floral organs, and employed a comparative RNA-Seq study to identify genes involved in Ag_2_S_2_O_3_-mediated sex modification and flower development. The study yielded a group of genes with potential roles in flower development and sex expression in Ag_2_S_2_O_3_ predisposed cannabis plants.

## Materials and methods

### Plant materials and silver thiosulfate treatment

Non-feminized seeds of licenced producer CanniMed Therapeutics line MS-17-338 were sown into moist peat/coco-fiber media in seed trays. After a month of growth, seedlings were transplanted into individual 2-gallon pots containing the peat/coco-fiber media, fertigated with inorganic NPK and micronutrients, under 18 h of light using 1000 W HPS and MH bulbs in separate proprietary reflectors. At 6–8 true leaf stage, small leaf cuttings were taken for DNA purification (GenElute Genomic Miniprep Kit Sigma-Aldrich), and PCR tests were run for male/female determination (Techen et al. 2010). There were four female and two male plants randomly chosen from an original 12 seedlings to grow to maturity. At 10 weeks of vegetative growth, lighting was switched to 12 h and a flowering NPK fertigation (decreased N and K and increased P) was initiated. At day one and three after starting 12 h of lighting and fertigation, two of the female plants were isolated for whole plant foliar application of 20 ml (2.5 µg/ml) of silver thiosulfate (Ag_2_S_2_O_3_) solution. This followed the method of Ram and Sett (1982), but replaced Tween-20 with Silwet^®^L 77 surfactant.

### *C. sativa* tissue samples and RNA isolation

**Fig. 1:**
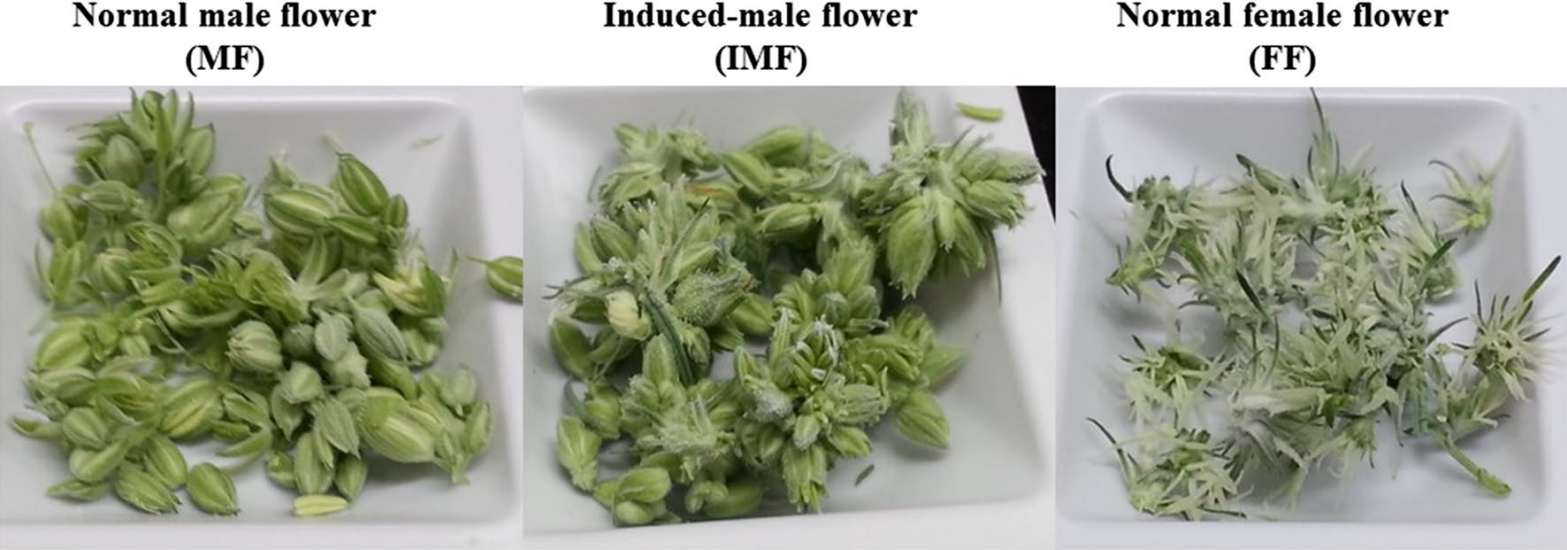
Floral bud samples of cannabis plants used for RNA-Seq studies between flower sex types. The flower sex types are normal male flower buds from genetically male plants; Ag_2_S_2_O_3_-induced male flower buds from genetically female plants; and normal female flower buds from genetically female plants. These flower buds were collected for RNA isolation at 14 days of post-light and fertigation induction of flowering

To develop a comprehensive combined transcriptome assembly, we sampled axillary shoot apical tissues prior to foliar Ag_2_S_2_O_3_ application (not shown in RNA-Seq study results). After 12 h light induction of flowering at day 0, foliar Ag_2_S_2_O_3_ application was made at days 1 and 3. Flower buds were sampled at days 8 and 14 post-flowering light induction. Samples of flower buds collected from each flower sex type at days 14 are shown in Fig. 1. The terminology refers to Ag_2_S_2_O_3_-induced-male flowers (IMF), normal female flowers (FF) and normal male plant (MF), used in differential gene expression studies. Axillary shoot apical tissue, and floral bud tissue samples were manually harvested and immediately flash-frozen in liquid nitrogen and stored at -80 °C until used. Frozen tissues (250 mg) of each sample were processed for total RNA purification using RNeasy Plant Mini Kit (Qiagen) according to the manufacturer’s protocol. Isolated RNA samples were then used for RNA-Seq and qPCR studies.

### RNA-Seq, de novo assembly and annotation

RNA sequencing was performed for a total of 15 samples (four for FF, five for MF and six for IMF) through a paid service at the DNA Sequencing Core Facility of The University of British Columbia (Vancouver, BC, Canada), using the Illumina NextSeq 500 via Paired-End (2 × 42 bp reads). All raw reads of these libraries have been deposited in the National Center for Biotechnology Information (NCBI) Sequence Read Archive (SRA) database under the BioProject accession PRJNA669389, BioSample accessions SAMN16447877–SAMN16447891, and SRA accessions SRR12831863–SRR12831877.

Raw RNA-Seq reads were processed using CLC Genomics Workbench v11.0 (Qiagen) for removing low-quality and short reads, trimming adaptors and generating paired reads. The resulting sets of paired reads were then assembled separately with respect to FF, MF and IMF using the CLC Genomics Workbench with a word size of 60. The three assemblies were combined and cleaned up (by removing the redundant sequences) using CD-HIT-EST (Li and Godzik 2006) with the threshold of 98% identity to generate a final reference transcriptome sequences. The transcriptome was validated by mapping the preprocessed paired reads back to the reference assembled sequences using CLC Genomics Workbench. The de novo-assembled reference transcript was then BLAST’ed against UniProKB database using tBLASTx with an e-value threshold of 0.001. We also BLAST’ed the assembled transcripts against Purple Kush reference transcriptome (van Bakel et al. 2011) under the same setup for further validation. The resulting UniProKB BLAST output was annotated and mapped with GO terms using the Blast2GO PRO plug-in for CLC Genomics Workbench.

### Differential expression analysis

Pre-processed RNA-Seq paired reads for each flower sex type (3 flower bud samples for FF, 4 flower bud samples for IMF and 2 flower bud samples for MF) were mapped to the final combined assembled transcriptome using the CLC Genomics Workbench. The expression levels of mapped paired reads were normalized as reads per kilobase per million mapped reads (RPKM) with the CLC RNA-Seq analysis tool. The normalized reads were then used for the differential expression analyses in three groups, including “IMF versus FF”, “IMF versus MF”, and “MF versus FF” using the same software. Differentially expressed genes (DEGs) were identified based on criteria set as an absolute log_2_ fold change ≥ 1, and a false discovery rate (FDR) *p *value ≤ 0.05. Differentially expressed genes were further characterized based on enriched metabolic pathways with MapMan (Usadel et al. 2009). The Mercator4 vs2.0 platform (Schwacke et al. 2019) was employed to annotate the DEG sequences with default settings to generate the mapping file that was used as an input in the pathway enrichment analysis in MapMan.

### Identification of DEGs linked to floral development and sex determination

Annotated DEGs homologous to genes involved in floral development and sex determination in other plants, including *Arabidopsis*, were manually screened using the CLC Genomics Workbench. Heatmap cluster analysis was performed for these genes with the MeV 4.9.0 program (Howe et al. 2011).

### qPCR analysis

qPCR analysis was performed for 15 DEGs selected from screened floral development and sex determination-linked genes using the same RNA samples that were used for RNA-Seq from each flower sex type. Briefly, RNA samples were reverse transcribed using iScript cDNA synthesis Kit (Bio-Rad) and used as templates in PCR assays. Two housekeeping genes (*actin: CsACT* and *elongation factor 2: CsEF2*) were used as reference genes for normalization. Quantitative PCR (qPCR) was carried out using the StepOne Plus Real-Time PCR system (Applied Bioscience) with a final reaction volume of 10 µl, consisting of 5 µl SYBR premix (Thermo Fisher), 0.6 µM of each primer and 150 ng of cDNA template. The amplification conditions were set withholding stage at 50 °C for 2 min, and 95 °C for 2 min, followed by 50 cycles at 95 °C for 3 s and 60 °C for 30 s, as well as a final melting curve stage at 95 °C for 15 s and 60 °C for 1 min. The primers used in this study are listed in Table S1.

### Statistical analyses

Using normalized RNA-Seq data (RPKM values), analyses of gene expression between flower sex types were performed using CLC Genome Workbench software, and the results were expressed as Fold Changes. The significance level of a differentially expressed gene (DEG) was determined based on its log_2_ fold change value, false discovery rate (FDR) p-value, and the corresponding mean value of RPKM (*n* = 2*–*4). If the gene has an RPKM mean of ≥ 5 at least in one of the flower sex type, and an absolute log_2_ fold change of ≥ 1, with an FDR p-value of ≤ 0.05, this gene is considered to be differentially expressed between the flower sex types. For validation, the expression patterns of selected genes were determined by qPCR using the $2^{{ - \Delta \Delta C_{{\text{t}}} }}$ method (Livak and Schmittgen 2001). Three technical replicates were performed for each of the two independent biological replicates. The mean value and standard error of qPCR data (log_2_ fold change; *n* = 2) of each gene and the corresponding mean and standard error value of RNA-Seq data (log_2_ RPKM; *n* = 2*–*4) were plotted together using SigmaPlot v 12.5 (Systat Software, Germany).

## Results

### Generation of masculinized female plants

To explore genes controlling sex determination, plants containing both female and male flower buds were developed from female plants after foliar treatments of female plants with silver thiosulfate complex. Flower buds on treated parts of the plants are hereafter referred to as induced-male flower (IMF). Three flower sex types, such as IMF, normal female flower (FF) and normal male flower (MF), were used for RNA isolation, from which a total of 15 samples (six for IMF, five for FF and four for MF) were sequenced to generate a combined comprehensive transcriptome sequence. The FF and MF samples were used as controls in RNA-Seq and qPCR analyses between flower sex types.

### RNA-Seq assembly and annotation

A total of 1,323,061,204 good quality paired reads were generated after the sequencing of 15 libraries from three different flower sex types (Table 1). The sequence samples for each flower sex type were separately de novo assembled and validated by mapping of paired reads against the assemblies. The resulting assemblies consisted of 40,615 transcripts in FF, 46,510 in IMF and 47,070 in MF, with N50 ranging from 1352 to 1564 bp (Table 2). After removing redundant sequences that had ≥ a 98% sequence identity, the three flower sex databases were combined, resulting in a total of 73,833 transcripts. The length distribution of these transcripts ranged from 200 to 14,620 bp, nearly 48% of which were 200–399 bp long (Fig. S1). Approximately 35% of these transcripts were also distributed between 400 and 1500 bp sizes, while the rest 17% were clustered under a size distribution of ≥ 1500 bp.

**Table 1:** RNA-Seq paired-read counts and alignment statistics for all samples used for de novo assembly of each flower sex type

Plant type	Sample name	# Paired reads	% Mapped paired reads	Type of tissues collected
Male flower (MF)	PPSMF1-1	100,792,342	90.65	Axillary shoot apical tissue
	PPSMF1-2	131,355,894	91.83	Axillary shoot apical tissue
	PPSMF3-1	94,509,194	89.52	Floral buds
	PPSMF3-2	67,307,650	91.44	Floral buds
Female flower (FF)	PPSFF1-1	88,935,852	89.52	Axillary shoot apical tissue
	PPSFF2E-1	85,206,482	89.22	Floral buds
	PPSFF2L-1	86,428,468	91.24	Floral buds
	PPSFF3-1	78,596,560	89.69	Floral buds
	PPSFF4-1	98,774,618	90.42	Mature flower
Induced-male flower (IMF)	PPSIMF1-1	56,195,344	89.84	Axillary shoot apical tissue
	PPSIMF1-2	109,023,982	91.59	Axillary shoot apical tissue
	PPSIMF2-1	74,871,936	85.97	Floral buds
	PPSIMF2-2	82,403,772	89.33	Floral buds
	PPSIMF3-1	98,449,940	91.23	Floral buds
	PPSIMF3-2	70,209,170	91.44	Floral buds
Total		1,323,061,204		

Over 1.3 billion good quality paired-end reads were generated from 15 sequencing libraries of three different flower sex types. The paired-end reads were successfully mapped back to the corresponding de novo assembled sequences, ranging from 89.5 to 91.8% in MF, 89.2 to 91.2% in FF, 86 to 91.6% in IMF

**Table 2:** Summary of assembly statistics for *C. sativa* flower transcripts from different flower sex types (contig measurement, including scaffold regions)

Parameters	Assembly statistics		
	Female flower (FF)	Induced-male flower (IMF)	Male flower (MF)
# contigs/transcripts	40,615	46,510	47,070
GC (%)	40.2	39.9	40.1
Average contig length (bp)	871	803	775
N50 (bp)	1564	1435	1352
Total assembled bases	35,379,806	37,366,991	36,488,663

The resulting assemblies consisted of 40,615 transcripts in FF, 46,510 in IMF and 47,070 in MF, with N50 ranging from 1352 to 1564 bp

Most of the assembled transcripts had BLAST hits against UniProtKB (62,350 transcripts; 85%) and cannabis Purple Kush transcriptome (63,696 transcripts; 86.3%) databases. Furthermore, the transcriptome included numerous full-length sequences, including those corresponding to genes of the MEP and MVA pathways of isoprenoid metabolism (Chen et al. 2011), previously known cannabis prenyltransferases and terpene synthases (Booth et al. 2017; Zager et al. 2019), and cannabinoid biosynthesis (Luo et al. 2019; Gülck and Møller 2020), indicating that the transcriptome adequately represents genes expressed in tissues used in this study.

Gene ontology (GO) terms were also determined for those annotated transcripts with BLAST hits. The top 20 GO category distribution for assembled transcripts in cellular component (CC), molecular functions (MF) and biological processes (BP) are shown in Fig. S2.

### Differential expression profiling

**Fig. 2:**
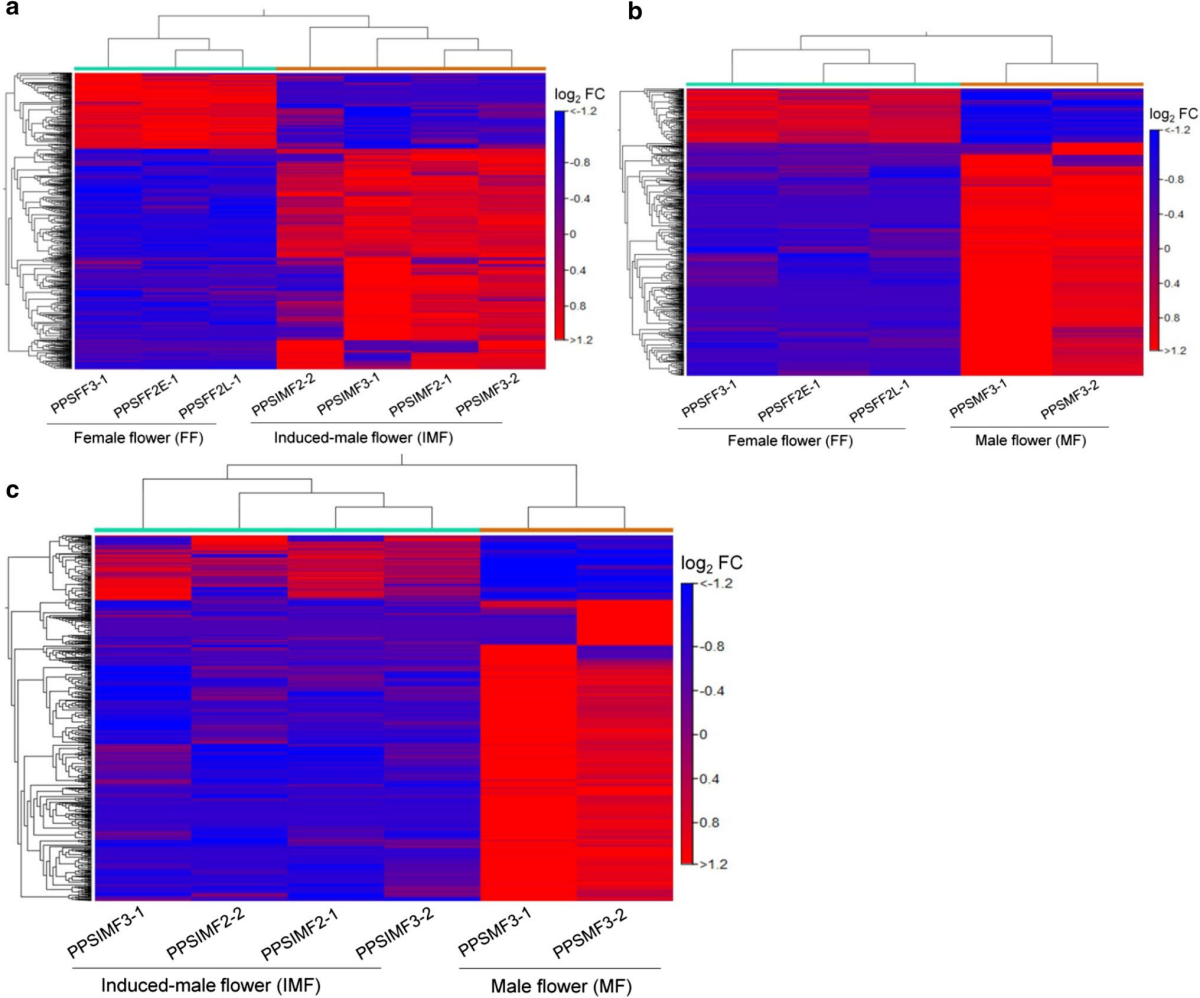
Hierarchical clustering of DEGs in different expression analysis assays. **a** IMFvsFF, **b** MFvsFF and **c** IMFvsMF libraries. Heatmaps show the relative expression levels of each transcript (row) in each flower sample (column). A comparable number of transcripts were differentially expressed in IMFvsFF (15%) and MFvsFF (17.6%) libraries, while slightly low numbers of DEGs (10%) were detected in IMFvsMF library, with the cut-off value of log_2_FC ≥ │1│and FDR ≤ 0.05. Expression values were normalized by RPKM and expressed as log_2_ fold change (log_2_FC), with a cut-off value of ≥ │1│and FDR *p *value ≤ 0.05. The color scale indicates upregulation (red) and downregulation (blue) of the transcripts in the samples

**Fig. 3:**
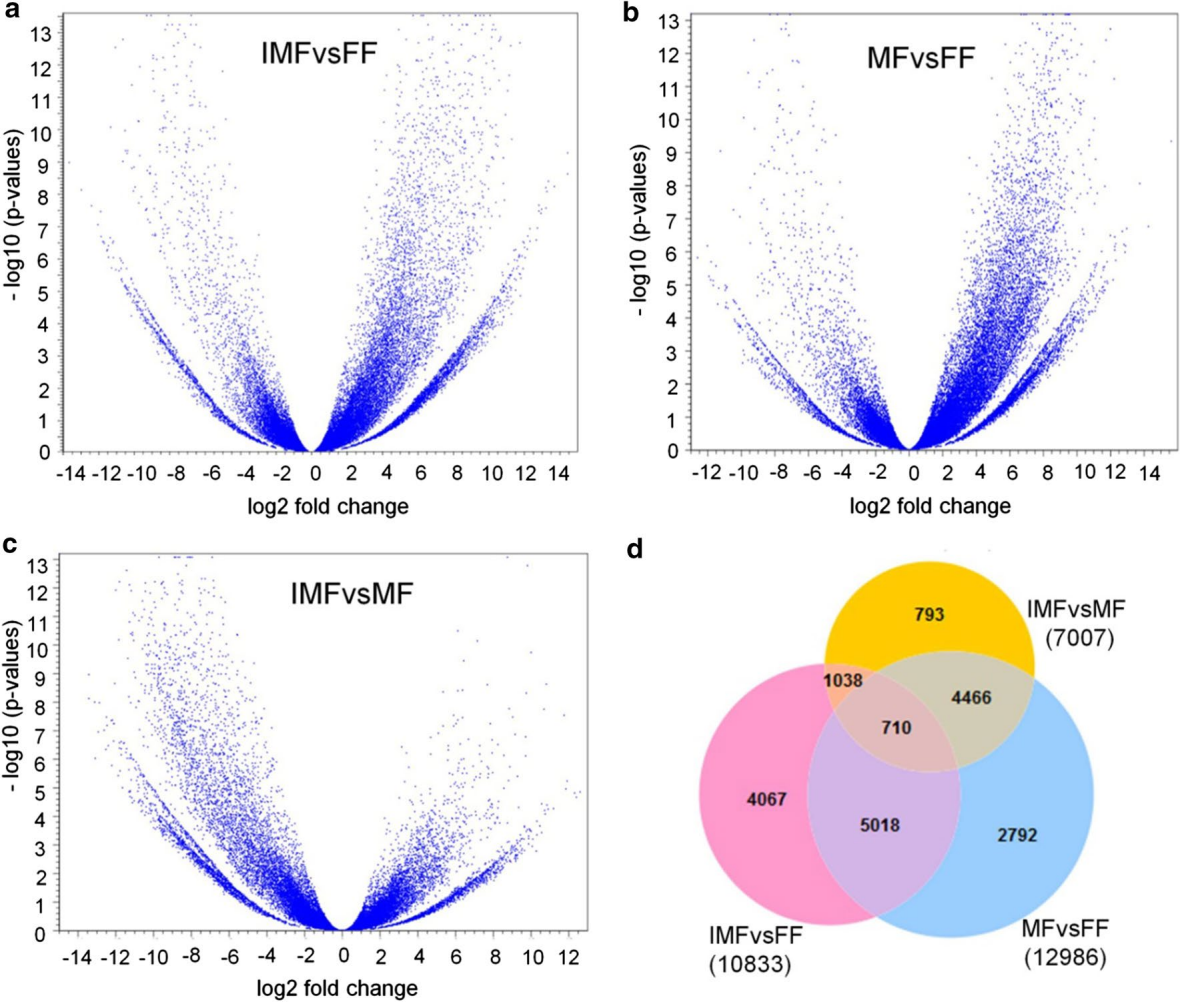
Expression of transcripts between cannabis floral sex types. Volcano plots displaying the expression of transcripts with false discovery rate (FDR) for **a** IMFvsFF, **b** MFvsFF, and **c** IMFvsMF. The log_10_(*p *values) indicates FDR *p* values. Transcripts with log_2_ fold change values of ≥ │1│ and FDR *p* value of ≤ 0.05 (–log_10_(*p* value) ≥ 1.30) are differentially expressed between the flower sex types. **d** Venn diagram showing the number of DEGs between cannabis flower sex types. With the cut-off value of log_2_FC ≥ │1│ and FDR ≤ 0.05, a total of 10,833 transcripts in IMFvsFF and of 12,986 transcripts in MFvsFF comparison were differentially expressed, from which nearly 50% of these DEGs were common in both comparisons. A total of 7007 transcripts were represented in the IMFvsMF comparison group, of which 793 (~ 11%) were differentially expressed only in this comparison

Using the assembled transcriptome as a reference sequence, transcript abundances for IMF and FF were compared to analyze the expression patterns. The comparison between transcripts of MFvsFF was made and used as a control for IMFvsFF comparison. A comparable number of transcripts were differentially expressed in IMFvsFF and MFvsFF libraries, accounting for about 15% (10,833) of the transcripts in IMFvsFF and 17.5% (12,986) in MFvsFF comparisons (log_2_FC ≥ │1│and FDR ≤ 0.05) (Figs. 2, 3). Among these differentially expressed transcripts, around 50% (5018) were expressed in both combinations, while the rest were specific to one of the libraries (Fig. 3). The majority of these expressed transcripts were found to be upregulated, ranging from 8085 in IMFvsFF to 10,561 transcripts in MFvsFF (Table 3). The top 500 DEGs were further compared between IMFvsFF and MFvsFF libraries based on metabolic pathway enrichments, resulting in similar expression patterns in most of the enriched pathways, including carbohydrate metabolism, lipid metabolism, phytohormone actions, cell wall organization, solute transport and external stimuli response (Fig. S3).

**Table 3:** Summary of differential expression levels across different sample libraries

Expression	log_2_ Fold Change (FC)	FDR *p *value	Number of transcripts		
			IMFvsFF	MFvsFF	IMFvsMF
Up-regulated	≥ 1	≤ 0.001	3003	4259	219
	≥ 1	0.001 < P ≤ 0.05	5082	6302	1025
	≥ 1	> 0.05	13,626	14,438	16,836
Not DEGs	│0 ≤ log_2_FC < 1│	–	32,957	27,793	34,937
	≤ − 1	> 0.05	14,158	16,374	12,831
Down-regulated	≤ − 1	≤ 0.001	778	559	2611
	≤ − 1	0.001 < P ≤ 0.05	2025	1904	3170
No data			2204	2204	2204
Total			73,833	73,833	73,833

The transcripts with log_2_FC values of  ≥ │1│ and FDR *p *value of ≤ 0.05 were considered to be differentially expressed. The expression levels of transcripts were categorized into upregulation (8085 in IMFvsFF, 10,561 in MFvsFF and 1244 in IMFvsMF), downregulation (2803 in IMFvsFF, 2463 in MFvsFF and 5781 in IMFvsMF), no differential expression (60,741 in IMFvsFF, 58,605 in MFvsFF and 64,604 in IMFvsMF) and no data (2204 in each comparison) between flower sex types based on log_2_ fold change (≤ − 1 or  ≥ 1) and FDR *p *values (≤ 0.05)

*FDR *false discovery rate, *FC* fold change; *DEGs *differentially expressed genes

In IMFvsMF library, we found a relatively low number of DEGs (7007), about 800 (11%) of which were uniquely expressed in this comparison (Fig. 3). The majority of these transcripts (4466, 64%) were differentially expressed in MFvsFF comparison, while only one-seventh (1038) of them were expressed in both comparison groups. We also analyzed male-specific transcripts that were not expressed in both genetically female background flowers, i.e., IMF and FF samples (mean RPKM = 0), and found a total of 180 transcripts, 30 of which had BLAST hits against UniProt1KB protein databases (Table S2).

### Identification of floral development and sex-determination related genes

**Fig. 4:**
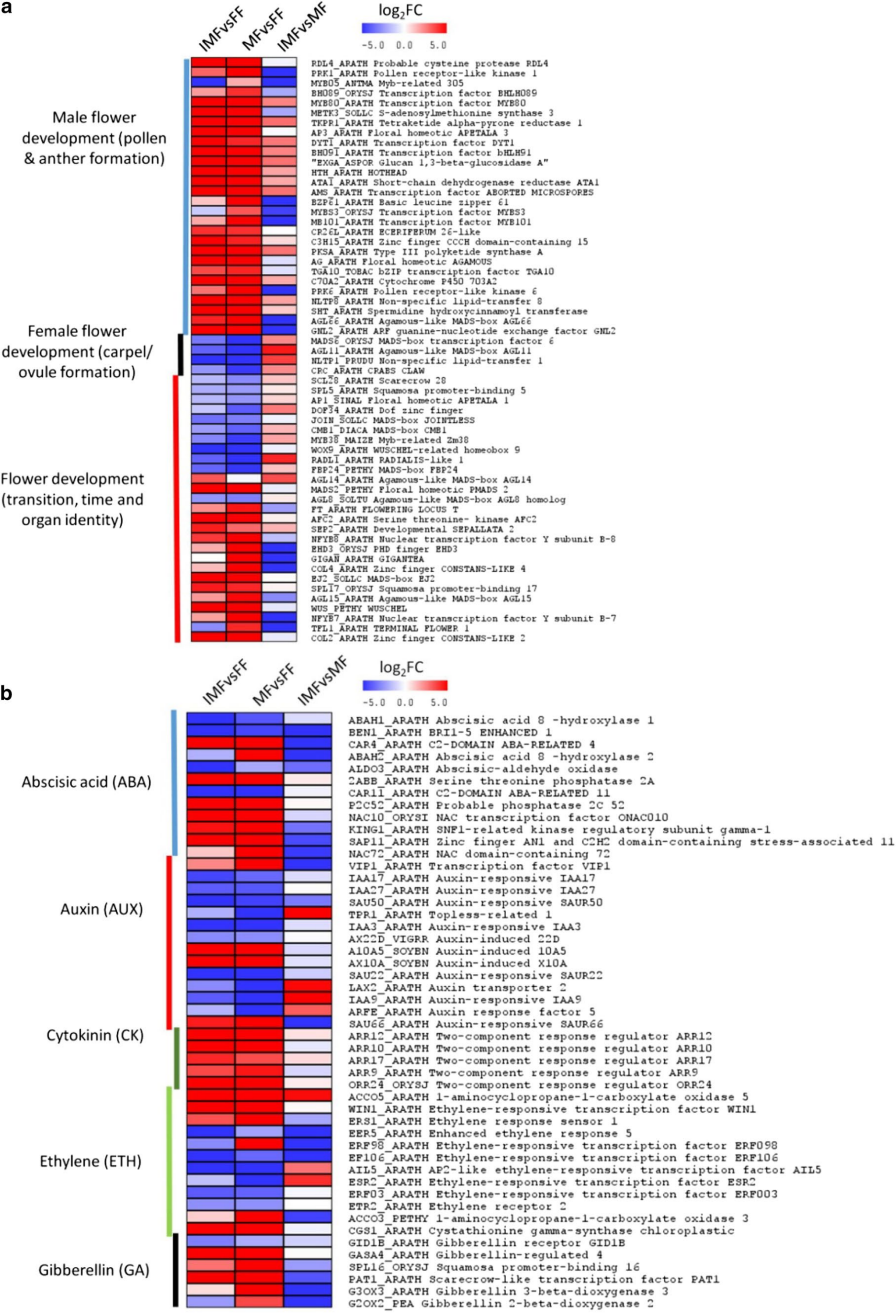
Heatmaps of key DEGs potentially controlling sex determination and floral development in IMFvsFF, MFvsFF and IMFvsMF libraries. **a** DEGs linked to floral development and sex determination. From a total of 245 DEGs, 61 DEGs (25%) potentially controlled anther/pollen development, floral transition and floral organ identity were identified in the three comparisons. More DEGs are involved in male flower regulations than those in female flowers. **b** DEGs associated with major hormone pathways and signalling. A total of 50 DEGs was identified in three comparisons. These DEGs are distributed to abscisic acid (ABA), auxin, cytokinin, ethylene, and gibberellin signalling pathways. The majority of the genes were differentially expressed in IMFvsFF and MFvsFF, while a few (19 DEGs) were detected from IMFvsMF. The relative expression of each gene (row) in each comparison (column) is shown. Expression values are log_2_ fold changes (log_2_FC) with color scales of red (upregulated) and blue (downregulated). MFvsFF library was used as a control for IMFvsFF comparison

To identify genes potentially involved in floral development and sex, we looked for genes that control these processes in other plants, and were differentially expressed in IMFvsFF, MFvsFF and IMFvsMF comparisons (Fig. 4 and Tables S3, S4 and S5). Over 81% (201) of these DEGs (245) were observed in the IMFvsFF comparison, 129 of which were highly expressed in induced-male flowers. The MFvsFF comparison revealed 207 DEGs, the majority (150 genes) of which were highly expressed in male flowers, while the other 57 genes were upregulated in female flowers. A total of 83 DEGs were detected in IMFvsMF comparison, 29 of which were highly represented in induced-male flowers, while the other 54 genes were abundant in male flowers.

The IMFvsFF and MFvsFF comparisons revealed 61 DEGs that potentially control anther/pollen development, floral transition and floral organ identity (Fig. 4a and Table S3). Some of the highly expressed transcripts associated with anther/pollen development in induced-male and natural male flowers include *Hothead (HTH), Type III polyketide synthase A (PKSA/LAP6), Tetraketide alpha-pyrone reductase 1 (TKPR1), Cytochrome P450 703A2 (C70A2), S-adenosylmethionine synthase 3 (METK3), APETALA 3 (AP3), ABORTED MICROSPORES (AMS), bHLH089, bHLH091, MYB80*, *DYT1* and *Spermidine hydroxycinnamoyl transferase (SHT)*. Compared to the male counterparts, the female flowers strongly expressed a few transcripts that are responsible for carpel/ovule development, apical meristem and other floral organ identities. These transcripts include *AGAMOUS-like MADS-box (AGL11)/SEEDSTICK (STK), MADS-box 6 (MADS6), Non-specific lipid-transfer 1 (NLTP1), Auxin-responsive IAA3 (IAA3)* and *WUSCHEL-related homeobox 9 (WOX9)*. In addition, 26 of these DEGs had shown differential expression in IMFvsMF comparison. A few of the highly expressed genes in IMF relative to MF include *Type III polyketide synthase A (PKSA/LAP6), Tetraketide alpha-pyrone reductase 1 (TKPR1), bHLH091* and *MYB80.*

A total of 50 transcripts that were differentially expressed in IMFvsFF and MFvsFF comparisons corresponded to abscisic acid (ABA) (13), auxin (13), cytokinin (CK) (5), ethylene (ETH) (12), gibberellin (GA) (6) and polyamine (1) signalling pathways (Fig. 4b and Table S4). For example, genes encoding *serine-threonine phosphatase 2A (2ABB*), probable *phosphatase 2C52 (P2C52)* and *SNF1-related kinase regulatory subunit gamma-1 (KING1)* in ABA, *auxin-induced X10A* and *auxin-induced 10A5* in auxin, *cystathionine gamma-synthase (CGS1), ethylene-responsive transcription factor WIN1* and *ethylene response sensor 1 (ERS1)* in ETH, and *gibberellin-regulated 4 (GASA4)* in GA were upregulated in induced-male and male flowers, compared to female flowers. Unlike other genes, transcripts corresponding to *auxin-responsive (IAA3), auxin-induced 22D (AX22D)* in auxin, and *ABA 8 hydroxylase 1, BRI1-5 ENHANCED 1, Abscisic-aldehyde oxidase* and *C2-domain ABA-related 11* in ABA were more strongly expressed in female flowers than in both male flower types. In induced-male flower, the transcripts homolog to the *two*-*component response regulator ARR9, ARR10, ARR12* and *ARR17* in CK and *1-aminocyclopropane-1-carboxylate oxidase 5 (ACCO5)* in ETH were upregulated, whereas *ethylene-responsive TFs 003 (ERF003) and ERF106* in ETH signalling were expressed at a lower level relative to female flowers. Moreover, only 19 of these DEGs were detected in the IMFvsMF comparison, and distributed to ABA (7), Auxin (5), GA (2) and ETH (5) (Fig. 4b and Table S3).

We further monitored highly expressed genes that are specifically associated with male flower development, cell wall and membrane formation, sugar and lipid metabolisms, phenylpropanoid and flavonoid biosynthesis, and other storage and transporters in IMFvsFF, MFvsFF and IMFvsMF comparisons (Table S5). The transcripts homologous to several male-specific genes including *MEN-8, cell division control 2 homolog (CDC2)* and *V-type proton ATPase subunit G1* were upregulated in induced-male and male flowers. Other transcripts-related to cell wall and plasma membrane formation (e.g., *xyloglucan endotransglucosylase hydrolase 23, Cellulose synthase A catalytic subunit 7, ATPase plasma membrane-type* and *Early nodulin 1*), transport (e.g., *Copper transporter 6 and Aquaporin NIP6-1*), sugar metabolism (e.g., *Mannose glucose-specific lectin* and *GDP-L-galactose phosphorylase 2*) and phenylpropanoid/flavonoid pathway (e.g., *Flavonol synthase* and *4-coumarate–ligase-like 1*) were among highly expressed genes in induced-male/male flowers, relative to female flowers. We also detected a few DEGs corresponding to these metabolic pathways between IMF and MF tissues. For example, there was a high expression of transcripts homolog to *Bifunctional dihydroflavonol 4-reductase flavanone 4-reductase* and *xyloglucan endotransglucosylase hydrolase 23,* and low expressions of *Lipid transfer EARLI 1, Sugar transport 10* and *Mini zinc finger 1* in IMF relative to MF.

### qPCR analysis

**Fig. 5:**
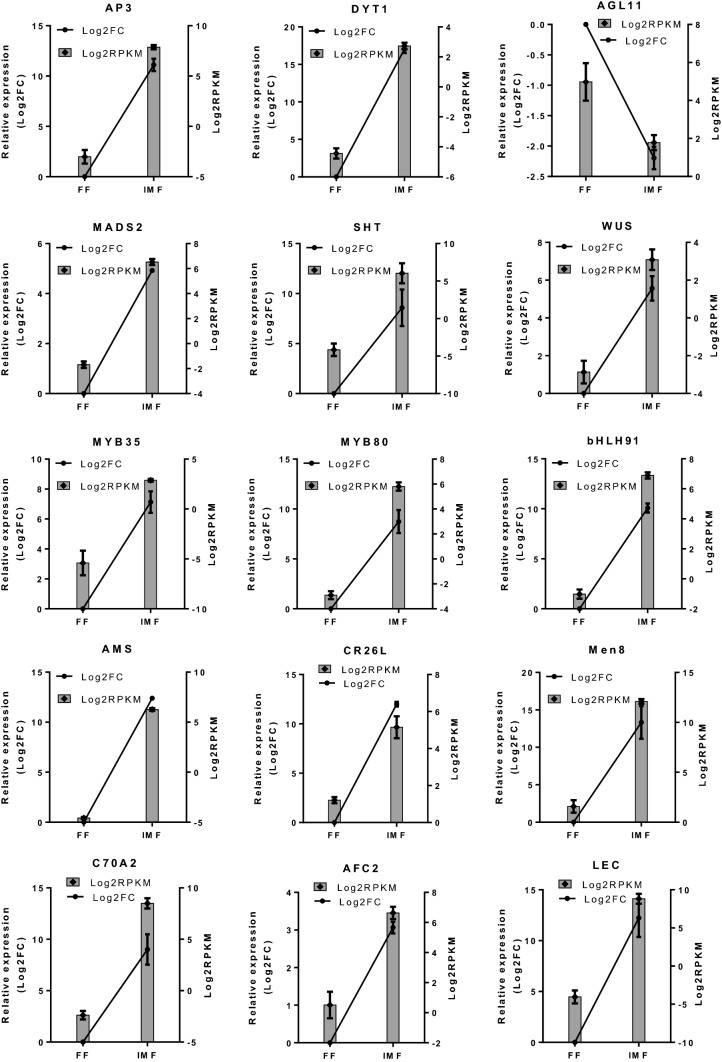
qPCR validation of selected DEGs in flowers of silver thiosulfate-induced male (IMF) and normal female (FF) plants. These DEGs include nine transcription factor genes: *APETALA 3 (AP3), Dysfunctional Tapetum1 (DYT1), Agamous-like MADS-box 11 (AGL11), MADS2, WUSCHEL (WUS), MYB35, MYB80, bHLH91* and *ABORTED MICROSPORES (AMS),* and six other genes: *Spermidine hydroxycinnamoyl transferase (SHT), Eceriferum 26-like (CR26), MEN-8 (male-specific protein—Men8), Cytochrome P450 703A2 (C70A2), Serine threonine- kinase AFC2 (AFC2)* and *Mannose glucose-specific lectin (LEC*) involved in floral development and sex determination. qPCR data were expressed as mean values ± standard errors (*n* = 2) of log_2_ fold change. The relative expression (qPCR) in FF samples was set arbitrary to 1 (log_2_(1) = 0). RNA-Seq data were shown as mean values ± standard errors (*n* = 2–4) of log_2_ RPKM. All of the targeted genes had similar expression patterns in both qPCR and RNA-Seq analyses between the two flower sex types

**Fig. 6:**
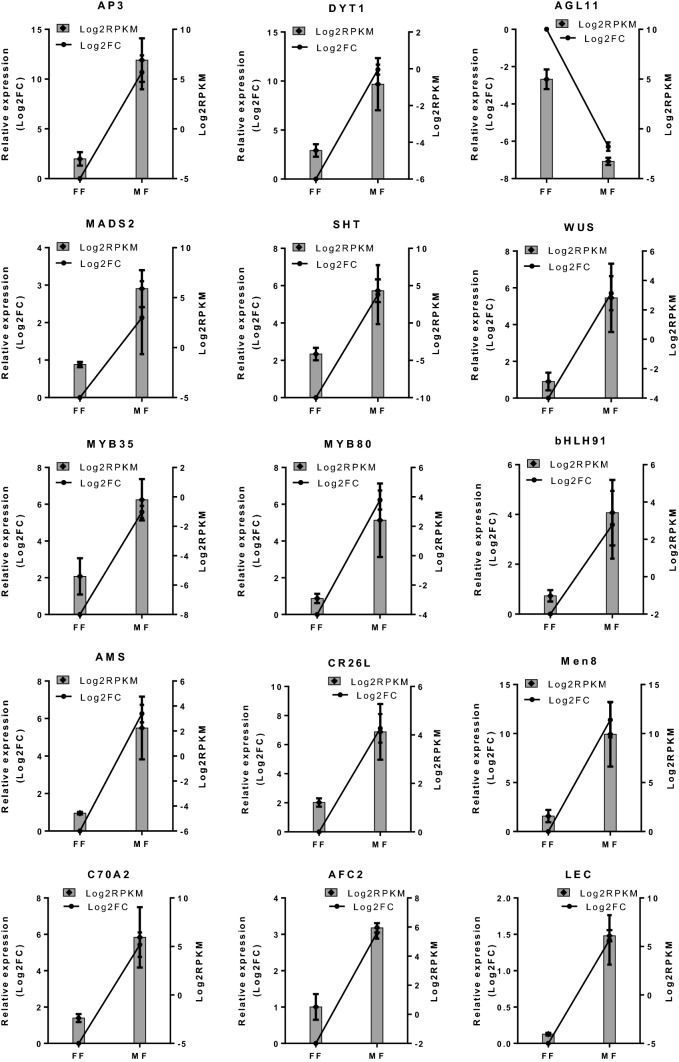
qPCR assays of selected DEGs in flowers of normal male (MF) and female (FF) plants. These DEGs include nine transcription factor genes: *APETALA 3 (AP3), Dysfunctional Tapetum1 (DYT1), Agamous-like MADS-box 11 (AGL11), MADS2, WUSCHEL (WUS), MYB35, MYB80, bHLH91*and *ABORTED MICROSPORES (AMS),* and six other genes: *Spermidine hydroxycinnamoyl transferase (SHT), Eceriferum 26-like (CR26), MEN-8 (male-specific protein—Men8), Cytochrome P450 703A2 (C70A2), Serine threonine- kinase AFC2 (AFC2)* and *Mannose glucose-specific lectin (LEC*) involved in floral development and sex determination. qPCR data were expressed as mean values ± standard errors (*n* = 2) of log_2_ fold change. The relative expression (qPCR) in the samples of FF was set arbitrary to 1 (log_2_(1) = 0). RNA-Seq data were shown as mean values ± standard errors (*n* = 2–4) of log_2_ RPKM. All of the targeted genes had similar expression patterns in both qPCR and RNA-Seq analyses between the two flower sex types

To validate the DEGs profiling based on RNA-Seq data, the expressions of 15 transcripts associated with floral development and sex determination were analyzed by a qPCR (Figs. 5, 6 and Fig. S4). These genes included nine transcription factors: *APETALA 3 (AP3), Dysfunctional Tapetum1 (DYT1), Agamous-like MADS-box 11 (AGL11), MADS2, WUSCHEL (WUS), MYB35, MYB80, bHLH91*and *ABORTED MICROSPORES (AMS),* and six other genes, including *Spermidine hydroxycinnamoyl transferase (SHT), Eceriferum 26-like (CR26), MEN-8 (male-specific protein -Men8), Cytochrome P450 703A2 (C70A2), Serine threonine- kinase AFC2 (AFC2)*and *Mannose glucose-specific lectin (LEC*). The generated normalized data from both qPCR and RNA-Seq were compared for each of the targeted genes in the IMFvsFF, MFvsFF and IMFvsMF comparisons. Both analysis approaches produced consistent expression patterns for all genes examined.

## Discussion

In *C. sativa*, the expression of plant sex is a complex process that is primarily controlled through the segregation of sex chromosomes (X and Y) during reproduction (Grant et al. 1994). However, epigenetic elements (e.g., DNA methylation and small regulatory RNA molecules), environmental cues (e.g., light and nutrients), plant hormones (e.g., auxin, gibberellins and abscisic acid), and chemical agents (e.g., silver thiosulfate and silver nitrate) can influence plant sex via as of yet unknown mechanisms (Ram and Jaiswal 1972; Galoch 1978; Ram and Sett 1982; Truta et al. 2007; Borthwick and Scully 1954). Foliar application of chemicals, such as silver thiosulfate or silver nitrate, can induce male organ development in genetically female background of several dioecious plant species, including *Asparagus officinalis, Ricinus communis, Coccinia grandis* and *C. Sativa* (Mohan Ram and Sett 1980; Ram and Sett 1982; Devani et al. 2017; Li et al. 2017). Although genomic and proteomic approaches have been used to examine chemical-based sexual expression in dioecious *A. officinalis* and *C. grandis* (Devani et al. 2017, 2019; Li et al. 2017), the molecular mechanism of this phenomenon remains unknown. In this study, we generated chemically induced male flower buds in genetically female *C. sativa* plants, and employed comparative RNA-Seq analyses among induced-male flowers, normal female flowers and normal male flowers to identify genes that mediate the expression of the opposite sex in predisposed female plants.

The RNA-Seq paired-reads were de novo assembled for each flower sex type, and combined to develop a comprehensive cannabis transcriptome of 73,833 transcripts. The assemblies contained most of the parameters that are comparable to those in transcriptomes previously reported for cannabis. For example, the N50 of the assemblies ranged from 1352 to 1564 bp, which are within the range of the previously published cannabis transcriptomes of Purple Kush (1804 bp), Finola (1193 bp) and Cannbio (1847 bp) (van Bakel et al. 2011; Grassa et al. 2018; Braich et al. 2019). The majority of transcripts were also annotated against UniProtKB and Purple Kush transcriptome, and assigned with GO terms. However, some of these transcripts were short (200–500 bp), which could be a reflection of parameters set for the CLC Genomics software. To produce a comprehensive transcriptome that maximizes our chances for gene discovery, including short length transcription factors, we set the parameter such that sequences of above 200 bp were included in the transcriptome. Regardless, the assembled transcriptome was of high quality as it contained full-length transcripts corresponding to previously reported genes related to the MEP and MVA pathways of isoprenoid metabolism (Chen et al. 2011), to cannabinoid biosynthesis (Luo et al. 2019; Gülck and Møller 2020), to terpene biosynthesis in *C. sativa* plants (Booth et al. 2017; Zager et al. 2019), and to flower development in other plants, e.g., *Arabidopsis* (Irish 2017).

Comparative transcriptomic analysis has been widely used between male and female flowers in a few dioecious plants, for example in *C. grandis* and *A. officinalis,* to explore sex-linked genes, or silver nitrate (AgNO_3_)-induced sex-biased genes that could play roles in flower sex determination (Devani et al. 2017; Li et al. 2017). A proteomic approach was also employed to identify proteins associated with AgNO_3_-induced sexual expression in *C. grandis* (Devani et al. 2019). These studies identified several transcripts linked to flower sex expression and floral development, including those in controlling aspects of plant hormone signaling. In this investigation, we employed a comparative transcriptomic analysis to identify genes that are potentially involved in flower sex modification in *C. sativa* plants. Several DEGs, including transcription factors and male organs-linked genes, which might play a role in promoting anther/pollen development in female plants, were identified. The development of sex-specific organs highly depends on the combinational expression of homeotic genes at different levels, time and space (Bouché et al. 2016; Wils and Kaufmann 2017). Among diverse homeotic genes, the B-class gene *APETALA 3 (AP3)* was strongly expressed in normal male/induced-male flowers, in which it could form a complex with another class B gene *PISTILLATA (PI)*, class C *AGAMOUS (AG)* and class E *SEPALLATA (SEP)* to specify the identity of stamens (Bouché et al. 2016; Wils and Kaufmann 2017). A few of the genes highly expressed in male and induced-male flowers (e.g., *Type III polyketide synthase A (LAP6/PKSA), Tetraketide alpha-pyrone reductase 1 (TKPR1)* and *Cytochrome P450 703A2 (C703A2))* are known to be involved in anther and pollen development (Morant et al. 2007; Kim et al. 2010; Xu et al. 2019). Similarly, some transcription factor homologs, including DYT1, bHLH89 and bHLH90, required for anther development (Zhu et al. 2015), and METK3 (S-adenosylmethionine synthase 3) involved in pollen tube growth (Chen et al. 2016), were more strongly expressed in male/induced-male flowers than female flowers. Another male-biased transcription factor AMS (ABORTED MICROSPORES) known to regulate tapetum development and pollen viability (Xu et al. 2010) was strongly expressed in male/induced-male flowers. The expression of most of these genes is consistent with those identified from dioecious *C. grandis* and *A. officinalis* upon AgNO_3_ application (Devani et al. 2017, 2019; Li et al. 2017), indicating that similar (at least partly) molecular mechanisms control flower sex modification in various predisposed plants.

It has been shown that genes involved in plant hormone biosynthesis and signalling are associated with flower sex determination in both monoecious and dioecious plants (Milewicz and Sawicki 2012; Devani et al. 2017, 2019; Li et al. 2017; Pawełkowicz et al 2019a, b). In monoecious plants (e.g., cucumber), the masculinizing and feminizing loci control similar hormone signalling pathways, and promote organ abortion processes (Mibus and Tatlioglu 2004; Martin et al. 2009). For example, ethylene suppresses male organ formation, and GA promotes the development of male parts in cucumber (Iwahori et al. 1970; Ando et al. 2001), in which these were supported by the expression of genes linked to the corresponding pathways (Zhang et al. 2017; Pawełkowicz et al 2019b). Likewise, the exogenous application of different hormones influences sexual phenotypes in cannabis. For instance, GA-treatment induces male flower, while auxin, ethylene and cytokinin promote female flowers in industrial hemp (Ram and Jaiswal 1972; Galoch 1978). In addition, ABA influences cannabis flower sex expression when applied in combination with other plant hormones (auxin or GA); ABA + IAA inhibited female flowers, while ABA + GA suppressed male flowers in hemp (Ram and Jaiswal 1972; Galoch 1978). On the other hand, it has been reported that silver ion (Ag^+^) inhibits ethylene action (Kumar et al. 2009; Yamasaki and Manabe 2011), and increases auxin efflux (Strader et al. 2009). The molecular basis of Ag^+^ action on flower sex is not known. In this study, we monitored the expression of transcripts associated with hormonal regulatory pathways among flower sex types, and detected several DEGs involved in the hormone signalling, including ethylene, auxin, GA, ABA and cytokinin. Ag^+^-induced male flowers strongly expressed transcripts homologous to ethylene signalling genes *cystathionine gamma-synthase (CGS1), 1-aminocyclopropane-1-carboxylate oxidase 5 (ACCO5)* and *ethylene response sensor 1 (ERS1)*. These flowers had low expression of transcripts corresponding to *ethylene-responsive TF 003* and *106 (ERF003* and *ERF106)* in ethylene signalling compared to female flowers. In contrast, the ethylene-related genes were not differentially expressed between normal male and female flowers. We also observed a high expression of a transcript homologous to the GA-related gene (*gibberellin-regulated 4 (GASA4))* in normal male/induced-male flowers, and low expressions of *auxin-responsive IAA3 (IAA3)* and *auxin-induced 22D (AX22D)* genes in female flowers. A few genes, including those encoding *serine-threonine phosphatase 2A (2ABB), probable phosphatase 2C 52 (P2C52)* and *SNF1-related kinase regulatory subunit gamma-1 (KING1)* involved in ABA signalling, were highly represented in normal male/induced-male flowers. Unlike normal male and female flower comparison, Ag^+^-induced male flowers had more expression of transcripts homolog to cytokinin signalling-related transcription factors, such as the *two*-*component response regulator ARR09, ARR10, ARR12* and *ARR17,* than female flowers. These findings imply that the levels of these hormones and their interactions might play critical roles in the regulation of cannabis flower sex determination. Furthermore, the results suggested that Ag^+^-induced phytohormone-mediated regulation may be useful for the targeted sex expression of cannabis plants.

We also found numerous DEGs homologous to those involved in cell wall and membrane formation, sugar/lipid metabolism, and phenylpropanoid and flavonoid biosynthesis between flower sex types. Similar comparative transcriptome analyses among Ag^+^-induced male, normal male and female flowers of dioecious asparagus and *C. grandis,* and monoecious cucumber have detected DEGs corresponding to cell wall/ membrane, sugar and lipid metabolisms, phenylpropanoid and flavonoid biosynthesis, transports and other pathways (Devani et al. 2017, 2019; Li et al. 2017; Pawełkowicz et al. 2019a, b). For example, cannabis flavonoid pathway gene homologs (e.g., *Flavonol synthase*) involved in pollen development and male sterility (Van Der Meer et al. 1992), and phenylpropanoid homology *4-coumarate-ligase-like 1* that can regulate flower development (Liu et al. 2017) were upregulated in male/induced-male flowers of cannabis.

Furthermore, we found a few transcripts specifically expressed in MF tissues compared to both IMF and FF samples. Although presumably a proposed X: autosome dosage mechanism (not genes on the Y chromosome) determines the cannabis sexual expression (Grant et al., 1994), the presence of these MF-specific transcripts is not surprising as genetically male-derived male flowers could express unique genes potentially located on the Y-chromosome. Overall, this study identified several DEGs that potentially control the masculinization of female cannabis plants. However, these DEGs may not be the only genes involved in this process, as this study was limited to tissue samples collected at mid to late stages of flower development. Additional regulatory genes presumably expressed during the early and late stages of flower development are also likely to contribute to flower sex expression, and should be further investigated.

## Conclusion

A fast-growing hemp farming industry particularly in North America and Europe is turning to marker-assisted breeding, SNP mapping and QTLs for advancing seed production and fiber quality for a multitude of applications including cannabinoid extraction for medicines. Along with the also expanding medical cannabis legislations and acceptance, the need for more insights into sex determination and flower development is rapidly progressing. As a step towards elucidation of the molecular basis of floral sex expression, we employed RNA-Seq to compare transcript abundances for genes expressed in male, female, and induced-male flower buds of *C. sativa* plants. The investigation highlighted a number of genes with potential roles in floral development and sex expression. Among these are genes homologous to those involved in flower development, and plant hormone signalling. The results suggest that silver thiosulfate-induced stamen development in female cannabis plants can be associated with complex networking of diverse genes involved in floral development, phytohormone signalling, sugar/lipid metabolism and other sex-related pathways. Although the exact roles of these genes must be further investigated in plants, for example, through overexpression and knockout experiments, the genes could be useful for the understanding of a plant’s predisposition to produce opposite sex flowers, and help growers to regulate this trait depending on the purpose of the cropping such as for seed and fiber or flower bud production for medicines.

### *Author contribution statement*

SSM and LH conceived and designed the research. LH and KD grew and treated the female plants with silver thiosulfate, and extracted RNA from different tissues. AMA and SSM conducted comparative RNA-Seq analyses and qPCR studies. AMA, SSM, LH and KD wrote the manuscript. All authors reviewed and approved the manuscript.

## Supplementary Information

Below is the link to the electronic supplementary material.

Supplementary file1 Fig. S1. The size distribution of total assembled transcripts from three cannabis flower sex types. The majority of the transcripts/ contigs were clustered into 200-400 bp in length. Fig. S2. The top 20 GO category distribution for assembled transcripts. Most of these transcripts were involved in cellular and metabolic processes under cellular component (CC), catalytic activity and binding under molecular function (MF) as well as cell and organelle under biological process (BP). Fig. S3. Summary of enriched MapMan metabolic pathways from the top 500 DEGs between flower tissues of cannabis sex type comparisons (IMFvsFF, MFvsFF and IMFvsMF). The metabolic pathway bins: 1- photosynthesis, 2- cellular respiration, 3-carbohydrate metabolism, 4- amino acid metabolism, 5- lipid metabolism, 6- nucleotide metabolism, 7- coenzyme metabolism, 8- polyamine metabolism, 9- secondary metabolism, 10- redox homeostasis, 11- phytohormone action, 12- chromatin organization, 13- cell cycle organization, 14- DNA damage response, 15- RNA biosynthesis, 16- RNA processing, 17- protein biosynthesis, 18- protein modification, 19- protein homeostasis, 20- cytoskeleton organization, 21- cell wall organization, 22- vesicle trafficking, 23- protein translocation, 24- solute transport, 25- nutrient uptake, 26- external stimuli response, and 35- unclassified proteins that can’t be assigned and/ or annotated. Each square represents a single DEG, and the log2 fold change (log2 FC) was used to generate the color scale varying from -4.5 (more red) to 4.5 (more blue). Dark blue color indicates higher expression in IMF or MF than FF, and dark red color shows more expression in FF compared to IMF or MF. White color indicates no differential expression between the plant flower sex types. More differentially expressed transcripts were observed in IMFvsFF and MFvsFF libraries than those in IMFvsMF. Fig. S4. qPCR validation of selected DEGs in flowers of IMF and MF plants. These DEGs include nine transcription factors: APETALA 3 (AP3), Dysfunctional Tapetum1 (DYT1), Agamous-like MADS-box 11 (AGL11), MADS2, WUSCHEL (WUS), MYB35, MYB80, bHLH91and ABORTED MICROSPORES (AMS), and six other genes: Spermidine hydroxycinnamoyl transferase (SHT), Eceriferum 26-like (CR26), MEN-8 (male-specific protein - Men8), Cytochrome P450 703A2 (C70A2), Serine threonine- kinase AFC2 (AFC2) and Mannose glucose-specific lectin (LEC) involved in floral development and sex determination. qPCR data were expressed as mean values ± standard errors (n= 2) of log2 fold change. The relative expression (qPCR) in the samples of FF was set arbitrary to 1 (log2(1)= 0). RNA-Seq data were shown as mean values ± standard errors (n= 2-4) of log2 RPKM. All of the targeted genes had similar expression patterns in both qPCR and RNA-Seq analyses between the two flower sex types. (DOCX 1111 KB)

Supplementary file2 Table S1. List of primers used for qPCR study. Table S2. Male-specific differentially expressed transcripts that contain BLAST hits against UniProtKB. A transcript is considered to be MF-specific, when it has a mean RPKM value of ≥ 5 in MF, but zero mean RPKM in both genetically female background FF and IMF samples. Table S3. Differentially expressed cannabis transcripts homologous to genes associated with floral development, including anther/pollen development & flower organ identity. A total of 245 sex determination and floral development associated DEGs were identified. Of these, 61 in the IMFvsFF and MFvsFF comparisons potentially control anther/ pollen development, floral transition and floral organ identity. A transcript is considered to be differentially expressed in a library, when it has a mean RPKM value of ≥ 5 at least in one of the two samples (e.g., IMF or FF in IMFvsFF library), with a log2 fold change value ≥│1│and FDR p-value ≤ 0.05. Table S4. Differentially expressed genes that are associated with phytohormone metabolism and signalling. These transcript homologs, which are potentially involved in flower sex determination and floral development, correspond to abscisic acid (13), auxin (13), cytokinin (6), ethylene (12), and gibberellin (6) signalling pathways. A transcript is considered to be differentially expressed in a library, when it has a mean RPKM value of ≥ 5 at least in one of the two samples (e.g., IMF or FF in IMFvsFF library), with a log2 fold change value ≥│1│and FDR p-value ≤ 0.05. Table S5. Differentially expressed genes related to sugar/lipid metabolisms, cell wall and membrane formations, and other male-specific genes. A transcript is considered to be differentially expressed in a library, when it has a mean RPKM value of ≥ 5 at least in one of the two samples (e.g., IMF or FF in IMFvsFF library), with a log2 fold change value ≥│1│and FDR p-value ≤ 0.05. (XLSX 69 KB)

## References

[CR1] Ando S, Sato Y, Kamachi S, Sakai S (2001). Isolation of a MADS-box gene (ERAF17) and correlation of its expression with the induction of formation of female flowers by ethylene in cucumber plants (*Cucumis sativus* L.). Planta.

[CR2] Bernstein N, Gorelick J, Koch S (2019). Interplay between chemistry and morphology in medical cannabis (*Cannabis sativa* L.). Ind Crops Prod.

[CR3] Booth JK, Page JE, Bohlmann J (2017). Terpene synthases from *Cannabis sativa*. PLoS ONE.

[CR4] Borthwick HA, Scully NJ (1954) Photoperiodic Responses of Hemp. Botanical gazette, 116:14–29. https://www.jstor.org/stable/2473219

[CR5] Bouché F, Lobet G, Tocquin P, Périlleux C (2016). FLOR-ID: an interactive database of flowering-time gene networks in *Arabidopsis thaliana*. Nucleic Acids Res.

[CR6] Braich S, Baillie RC, Jewell LS (2019). Generation of a comprehensive transcriptome atlas and transcriptome dynamics in medicinal cannabis. Sci Rep.

[CR7] Causier B, Schwarz-Sommer Z, Davies B (2010). Floral organ identity: 20 years of ABCs. Semin Cell Dev Biol.

[CR8] Chen F, Tholl D, Bohlmann J, Pichersky E (2011). The family of terpene synthases in plants: a mid-size family of genes for specialized metabolism that is highly diversified throughout the kingdom. Plant J.

[CR9] Chen Y, Zou T, McCormick S (2016). S-adenosylmethionine synthetase 3 is important for pollen tube growth. Plant Physiol.

[CR10] Devani RS, Sinha S, Banerjee J (2017). De novo transcriptome assembly from flower buds of dioecious, gynomonoecious and chemically masculinized female *Coccinia grandis* reveals genes associated with sex expression and modification. BMC Plant Biol.

[CR11] Devani RS, Chirmade T, Sinha S (2019). Flower bud proteome reveals modulation of sex-biased proteins potentially associated with sex expression and modification in dioecious *Coccinia grandis*. BMC Plant Biol.

[CR12] Divashuk MG, Alexandrov OS, Razumova OV (2014). Molecular cytogenetic characterization of the dioecious *Cannabis sativa* with an XY chromosome sex determination system. PLoS ONE.

[CR13] Galoch E (1978). The hormonal control of sex differentiation in dioecious plants of hemp (*Cannabis sativa*): the influence of plant growth regulators on sex expression in male and female plants. Acta Soc Bot Pol.

[CR14] Gao Y, Gao Y, Fan M (2017). Overexpression of Chrysanthemum morifolium SVP gene delays blossoming and regulates inflorescence architecture in transgenic Arabidopsis. Can J Plant Sci.

[CR15] Grant S, Houben A, Vyskot B, Siroky J, Pan WH, Macas J, Saedler H (1994). *Genetics* of sex determination in flowering plants. Dev Genet.

[CR16] Grassa CJ, Wenger JP, Dabney C (2018). A complete Cannabis chromosome assembly and adaptive admixture for elevated cannabidiol (CBD) content. bioRxiv.

[CR17] Gülck T, Møller BL (2020). Phytocannabinoids: origins and biosynthesis. Trends Plant Sci.

[CR18] Howe EA, Sinha R, Schlauch D, Quackenbush J (2011). RNA-Seq analysis in MeV. Bioinform Appl Notes.

[CR19] Irish V (2017). The ABC model of floral development. Curr Biol.

[CR20] Iwahori S, Lyons JM, Smith OE (1970). Sex expression in cucumber plants as affected by 2-chloroethylphosphonic acid, ethylene, and growth regulators. Plant Physiol.

[CR60] Kater MM, Colombo L, Franken J, Busscher M, Masiero S, Van Lookeren Campagne MM, Angenent GC (1998). Multiple homologs from cucumber and petunia differ in their ability to induce reproductive organ fate. Plant Cell.

[CR21] Kim SS, Grienenberger E, Lallemand B (2010). LAP6/POLYKETIDE SYNTHASE A and LAP5/POLYKETIDE SYNTHASE B encode hydroxyalkyl α-pyrone synthases required for pollen development and sporopollenin biosynthesis in *Arabidopsis thaliana*. Plant Cell.

[CR22] Kram BW, Xu WW, Carter CJ (2009). Uncovering the arabidopsis thaliana nectary transcriptome: Investigation of differential gene expression in floral nectariferous tissues. BMC Plant Biol.

[CR23] Kumar V, Parvatam G, Ravishankar GA (2009). AgNO_3_—a potential regulator of ethylene activity and plant growth modulator. Electron J Biotechnol.

[CR24] Li W, Godzik A (2006). Cd-hit: a fast program for clustering and comparing large sets of protein or nucleotide sequences. Bioinformatics.

[CR26] Li SF, Zhang GJ, Zhang XJ (2017). Comparative transcriptome analysis reveals differentially expressed genes associated with sex expression in garden asparagus (*Asparagus officinalis*). BMC Plant Biol.

[CR27] Litt A, Kramer EM (2010). The ABC model and the diversification of floral organ identity. Semin Cell Dev Biol.

[CR28] Liu H, Guo Z, Gu F (2017). 4-Coumarate-CoA ligase-like gene OsAAE3 negatively mediates the rice blast resistance, floret development and lignin biosynthesis. Front Plant Sci.

[CR29] Livak KJ, Schmittgen TD (2001). Analysis of relative gene expression data using real-time quantitative PCR and the 2^(-ΔΔCT) method. Methods.

[CR30] Luo X, Reiter MA, d’Espaux L (2019). Complete biosynthesis of cannabinoids and their unnatural analogues in yeast. Nature.

[CR31] Martin A, Troadec C, Boualem A (2009). A transposon-induced epigenetic change leads to sex determination in melon. Nature.

[CR32] Mibus H, Tatlioglu T (2004). Molecular characterization and isolation of the F/f gene for femaleness in cucumber (*Cucumis sativus* L.). Theor Appl Genet.

[CR33] Milewicz M, Sawicki J (2012). Mechanisms of sex determination in plants. Acta Musei Silesiae, Sci Nat.

[CR34] Mohan Ram HY, Sett R (1980). Induction of male flowers in a pistillate line of *Ricinus communis* L. by silver and cobalt ions. Planta.

[CR59] Mohan Ram HY, Nath R (1964). The morphology and embryology of Cannabis sativa L. Phytomorphology.

[CR35] Moliterni VMC, Cattivelli L, Ranalli P, Mandolino G (2004). The sexual differentiation of *Cannabis sativa* L.: a morphological and molecular study. Euphytica.

[CR36] Morant M, Schaller H, Pinot F (2007). CYP703 is an ancient cytochrome P450 in land plants catalyzing in-chain hydroxylation of lauric acid to provide building blocks for sporopollenin synthesis in pollen. Plant Cell.

[CR38] Pawełkowicz M, Pryszcz L, Skarzynska A (2019). Comparative transcriptome Analysis reveals new molecular pathways for cucumber genes related to sex determination. Plant Reprod.

[CR39] Pawełkowicz M, Skarzynska A, Plader W (2019). Genetic and molecular bases of cucumber (*Cucumis sativus* L.) sex determination. Mol Breed.

[CR40] Ram HYM, Jaiswal VS (1972). Induction of male flowers on female plants of *Cannabis sativa* by gibberellins and its inhibition by abscisic acid. Planta.

[CR41] Ram HYM, Sett R (1982). Induction of fertile male flowers in genetically female *Cannabis sativa* plants by silver nitrate and silver thiosulphate anionic complex. Theor Appl Genet.

[CR42] Rana A, Choudhary N (2010). Floral biology and pollination biology of *Cannabis sativa* L. Int J Plant Reprod Biol.

[CR43] Schwacke R, Ponce-Soto GY, Krause K (2019). MapMan4: a refined protein classification and annotation framework applicable to multi-omics data analysis. Mol Plant.

[CR44] Searle I, Coupland G (2004). Induction of flowering by seasonal changes in photoperiod. EMBO J.

[CR45] Strader LC, Beisner ER, Bartel B (2009). Silver ions increase auxin efflux independently of effects on ethylene response. Plant Cell.

[CR61] Techen N, Chandra S, Lata H, ElSohly M, Khan I (2010). Genetic identification of female plants at early developmental stage. Planta Medica.

[CR46] Thomas BF, ElSohly MA (2016). Biosynthesis and pharmacology of phytocannabinoids and related chemical constituents. Anal Chem Cannabis.

[CR47] Truta E, Olteanu Z, Surdu S, et al. (2007) Some aspects of sex determinism in hemp. Analele Stiint ale Univ Alexandru Ioan Cuza" din Iasi Sec II. a Genet si Biol Mol 8:31–39

[CR48] Usadel B, Poree F, Nagel A (2009). A guide to using MapMan to visualize and compare Omics data in plants: a case study in the crop species, Maize. Plant Cell Environ.

[CR49] Van Bakel H, Stout JM, Cote AG (2011). The draft genome and transcriptome of *Cannabis sativa*. Genome Biol.

[CR50] Van Der Meer IM, Stam ME, Van Tunen AJ (1992). Antisense inhibition of flavonoid biosynthesis in petunia anthers results in male sterility. Plant Cell.

[CR51] Wells RS, Adal AM, Bauer L (2020). Cloning and functional characterization of a floral repressor gene from *Lavandula angustifolia*. Planta.

[CR52] Wils CR, Kaufmann K (2017). Gene-regulatory networks controlling in fl orescence and fl ower development in *Arabidopsis thaliana*. Biochem Biophys Acta.

[CR53] Xu J, Yang C, Yuan Z (2010). The ABORTED MICROSPORES regulatory network is required for postmeiotic male reproductive development in *Arabidopsis thaliana*. Plant Cell.

[CR54] Xu D, Qu S, Tucker MR (2019). Ostkpr1 functions in anther cuticle development and pollen wall formation in rice. BMC Plant Biol.

[CR55] Yamasaki S, Manabe K (2011). Application of silver nitrate induces functional bisexual flowers in gynoecious cucumber plants (*Cucumis sativus* L.). J Japanese Soc Hortic Sci.

[CR56] Zager JJ, Lange I, Srividya N (2019). Gene networks underlying cannabinoid and terpenoid accumulation in cannabis. Plant Physiol.

[CR57] Zhang Y, Zhao G, Li Y (2017). Transcriptomic analysis implies that GA regulates sex expression via ethylene-dependent and ethylene-independent pathways in cucumber (*Cucumis sativus* L.). Front Plant Sci.

[CR58] Zhu E, You C, Wang S (2015). The DYT1-interacting proteins bHLH010, bHLH089 and bHLH091 are redundantly required for Arabidopsis anther development and transcriptome. Plant J.

