# Supplementary figures and images for: Comparative RNA-Seq analysis reveals genes associated with masculinization in female Cannabis sativa
Source: Planta. 2021 Jan 4;253(1):17. doi: 10.1007/s00425-020-03522-y (PMC7779414; doi:10.1007/s00425-020-03522-y)

Fig. S1


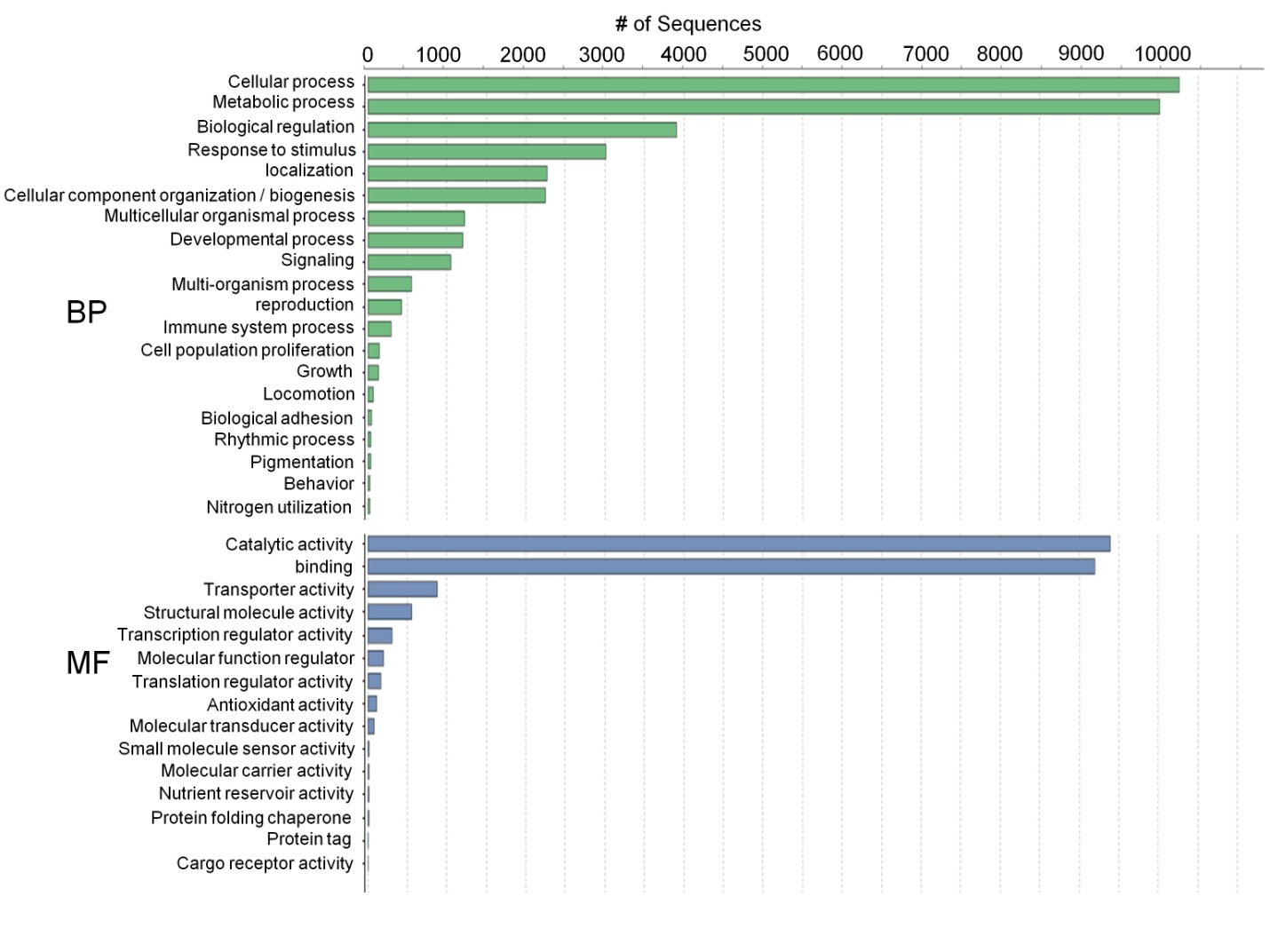

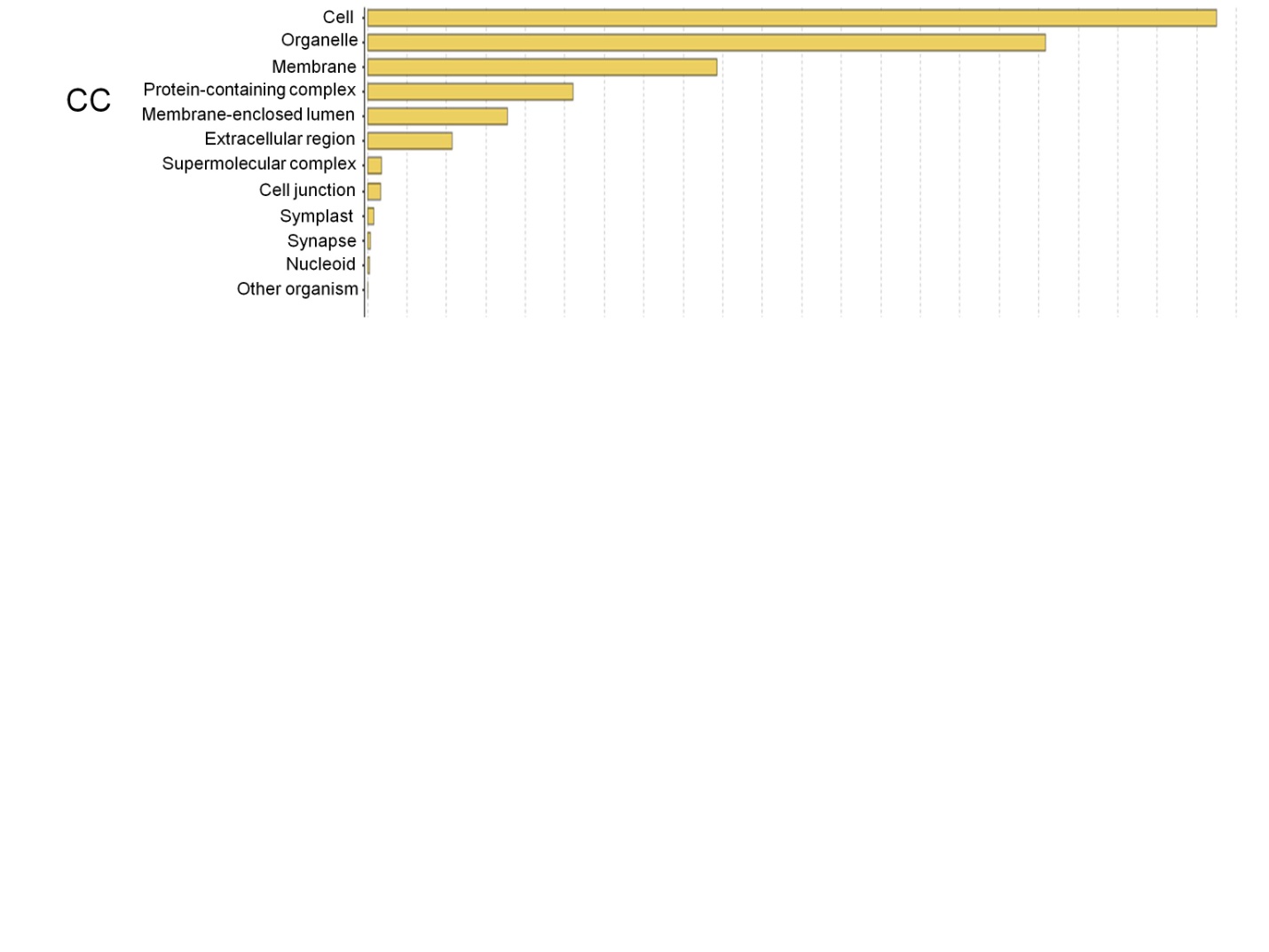


Fig. S2


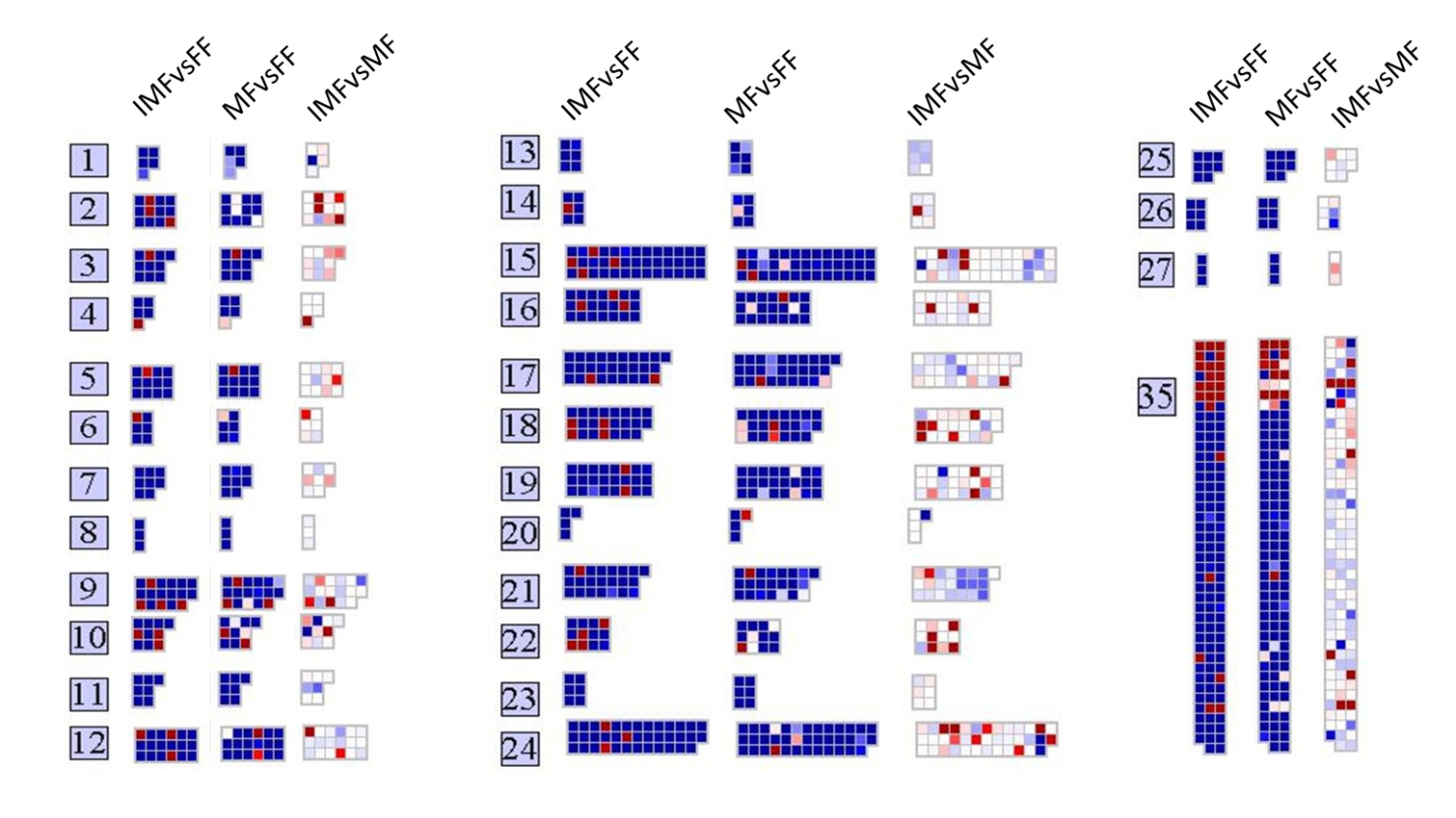


Fig. S3.

Fig. S4.

Supplement: Supplementary file 1 — Supplementary file1 Fig. S1. The size distribution of total assembled transcripts from three cannabis flower sex types. The majority of the transcripts/ contigs were clustered into 200-400 bp in length. Fig. S2. The top 20 GO category distribution for assembled transcripts. Most of these transcripts were involved in cellular and metabolic processes under cellular component (CC), catalytic activity and binding under molecular function (MF) as well as cell and organelle under biological process (BP). Fig. S3. Summary of enriched MapMan metabolic pathways from the top 500 DEGs between flower tissues of cannabis sex type comparisons (IMFvsFF, MFvsFF and IMFvsMF). The metabolic pathway bins: 1- photosynthesis, 2- cellular respiration, 3-carbohydrate metabolism, 4- amino acid metabolism, 5- lipid metabolism, 6- nucleotide metabolism, 7- coenzyme metabolism, 8- polyamine metabolism, 9- secondary metabolism, 10- redox homeostasis, 11- phytohormone action, 12- chromatin organization, 13- cell cycle organization, 14- DNA damage response, 15- RNA biosynthesis, 16- RNA processing, 17- protein biosynthesis, 18- protein modification, 19- protein homeostasis, 20- cytoskeleton organization, 21- cell wall organization, 22- vesicle trafficking, 23- protein translocation, 24- solute transport, 25- nutrient uptake, 26- external stimuli response, and 35- unclassified proteins that can’t be assigned and/ or annotated. Each square represents a single DEG, and the log2 fold change (log2 FC) was used to generate the color scale varying from -4.5 (more red) to 4.5 (more blue). Dark blue color indicates higher expression in IMF or MF than FF, and dark red color shows more expression in FF compared to IMF or MF. White color indicates no differential expression between the plant flower sex types. More differentially expressed transcripts were observed in IMFvsFF and MFvsFF libraries than those in IMFvsMF. Fig. S4. qPCR validation of selected DEGs in flowers of IMF and MF plants. These DEGs incl [file 425_2020_3522_MOESM1_ESM.docx]
